# GETTING OLDER AND GETTING BY WITH SSI

**DOI:** 10.1093/geroni/igae098.2506

**Published:** 2024-12-31

**Authors:** Callie Freitag

**Affiliations:** University of Wisconsin-Madison, Wisconsin, United States

## Abstract

For decades, qualitative researchers have asked low-income younger adults how they make ends meet in light of a shifting safety net. Yet, we know comparatively little about the daily economic lives of an increasingly policy-relevant group: low-income older adults who receive Supplemental Security Income (SSI). SSI provides a monthly backstop of cash assistance, a maximum of $943 per month in 2024, but its strict income and asset limits may mitigate its poverty-reducing potential. Additional disability-based eligibility requirements for people under age 65 may create administrative burden and perpetuate economic and health disparities. This project uses nationally representative, secondary qualitative interview data from the American Voices Project to examine the life events that led older adults to take up SSI and describe how they make sense of their economic circumstances in light of SSI’s benefits and constraints. From a stratified subsample of low-income adults ages 50 years older (N=28), I take an abductive approach to analyze interviews and compare findings across respondents who entered SSI through the disability and retirement pathways. I find that a wide array of life experiences lead older adults to SSI, but nearly all are underscored by a lifetime of precarious employment and persistent poverty. Most respondents say they are able to “get by” between their monthly SSI check and other resources like subsidized housing, but are unable to cope with large, unexpected expenses without turning to family or debt.

